# Chlorite oxidized oxyamylose differentially influences the microstructure of fibrin and self assembling peptide hydrogels as well as dental pulp stem cell behavior

**DOI:** 10.1038/s41598-021-84405-4

**Published:** 2021-03-11

**Authors:** Mostafa EzEldeen, Burak Toprakhisar, Denise Murgia, Nick Smisdom, Olivier Deschaume, Carmen Bartic, Hans Van Oosterwyck, Rafaela Vaz Sousa Pereira, Ghislain Opdenakker, Ivo Lambrichts, Annelies Bronckaers, Reinhilde Jacobs, Jennifer Patterson

**Affiliations:** 1 OMFS IMPATH Research Group, Faculty of Medicine, Department of Imaging and Pathology, KU Leuven and Oral and Maxillofacial Surgery, University Hospitals Leuven, Kapucijnenvoer 33, 3000 Leuven, Belgium; 2 Department of Oral Health Sciences, KU Leuven and Paediatric Dentistry and Special Dental Care, University Hospitals Leuven, Kapucijnenvoer 33, 3000 Leuven, Belgium; 3 Stem Cell Institute, KU Leuven, University of Leuven, Leuven, Belgium; 4 Department of Surgical, Oncological and Oral Sciences, University of Palermo, Via Liborio Giuffrè 5, 90127 Palermo, Italy; 5 Biomedical Research Institute, Hasselt University, Campus Diepenbeek, Agoralaan Building C, 3590 Diepenbeek, Belgium; 6 Soft-Matter Physics and Biophysics Section, KU Leuven, University of Leuven, Leuven, Belgium; 7 Biomechanics Section, Department of Mechanical Engineering, KU Leuven, University of Leuven, Leuven, Belgium; 8 Department of Microbiology, Immunology and Transplantation, Rega Institute for Medical Research, KU Leuven, University of Leuven, Leuven, Belgium; 9 Department of Dental Medicine, Karolinska Institute, Stockholm, Sweden; 10 IMDEA Materials Institute, Tecnogetafe, Calle Eric Kandel, 2, 28906 Getafe, Madrid Spain; 11 Prometheus Division of Skeletal Tissue Engineering, KU Leuven, University of Leuven, Leuven, Belgium

**Keywords:** Endodontics, Biomaterials, Dentistry, Dental biomaterials, Mesenchymal stem cells

## Abstract

Tailored hydrogels mimicking the native extracellular environment could help overcome the high variability in outcomes within regenerative endodontics. This study aimed to evaluate the effect of the chemokine-binding and antimicrobial polymer, chlorite-oxidized oxyamylose (COAM), on the microstructural properties of fibrin and self-assembling peptide (SAP) hydrogels. A further goal was to assess the influence of the microstructural differences between the hydrogels on the in vitro behavior of human dental pulp stem cells (hDPSCs). Structural and mechanical characterization of the hydrogels with and without COAM was performed by atomic force microscopy and scanning electron microscopy to characterize their microstructure (roughness and fiber length, diameter, straightness, and alignment) and by nanoindentation to measure their stiffness (elastic modulus). Then, hDPSCs were encapsulated in hydrogels with and without COAM. Cell viability and circularity were determined using confocal microscopy, and proliferation was determined using DNA quantification. Inclusion of COAM did not alter the microstructure of the fibrin hydrogels at the fiber level while affecting the SAP hydrogel microstructure (homogeneity), leading to fiber aggregation. The stiffness of the SAP hydrogels was sevenfold higher than the fibrin hydrogels. The viability and attachment of hDPSCs were significantly higher in fibrin hydrogels than in SAP hydrogels. The DNA content was significantly affected by the hydrogel type and the presence of COAM. The microstructural stability after COAM inclusion and the favorable hDPSCs' response observed in fibrin hydrogels suggest this system as a promising carrier for COAM and application in endodontic regeneration.

## Introduction

Oral health plays an essential role in our daily lives, contributing to good overall health and wellbeing. Yet, impaired oral conditions have a high prevalence, affecting almost half of the world population^1^. Dental pulp necrosis due to caries, trauma, or developmental anomalies is standardly treated by filling the root canal space with bio-inert plastic-like materials, thus depriving the tooth of vascularization, an immune response, and innervation. Immature teeth with pulp necrosis are rendered fragile even after treatment, and the roots fail to reach complete development.

While the classical regenerative medicine approach employs a combination of cells with biomaterials to promote tissue regeneration, the economic and regulatory hurdles associated with cell-based therapies have also led to the proliferation of cell-free biomaterial approaches, which stimulate the activity of endogenous stem cells. Therefore, regenerative endodontics can be approached either in a cell-free or cell-based manner^2–4^ and has attracted attention attempting to restore tooth vitality^5^. A cell-free clinical protocol intended to reestablish the pulp-dentin complex has been developed, which is known under the synonyms pulp revitalization, root-canal revascularization, or regenerative endodontic treatment (RET)^6–8^. Nevertheless, RET has been associated with highly variable outcomes^9–11^, and histologic studies have shown that true pulp regeneration using the current protocol is challenging to achieve^7,11,12^.

Furthermore, a cell-based RET approach would utilize human dental pulp stem cells (hDPSCs), stem cells from human exfoliated deciduous teeth (SHED), or stem cells of the apical papilla (SCAPs). hDPSCs, SHED, and SCAPs are mesenchymal stem/stromal cells that possess the potential to differentiate into numerous cell types in vitro*,* including odontoblasts, osteoblasts, chondroblasts, adipocytes, and neuronal cells^13–15^. The relative ease of accessibility from extracted wisdom molars or exfoliated primary teeth^16^ renders them a valuable tool for studying and exploring tissue regeneration possibilities in the dentoalveolar and craniofacial regions.

Polymeric hydrogels are suitable candidates for tissue engineering and regenerative medicine (TERM) approaches, including dental pulp regeneration^17^. The use of tailored hydrogels closely mimicking the native extracellular environment could help overcome the high variability in the RET outcomes. Many recent studies have demonstrated that cell behavior is strongly influenced by the cell microenvironment^18^, which is dictated by the hydrogels' composition and microstructure^19,20^. Polymeric hydrogels can be natural (biopolymers), synthetic, or hybrids of the two^21,22^, with several advantages and disadvantages related to each class^23^. Fibrin is a typical natural hydrogel, and it has been extensively used as a biomaterial for different TERM and clinical applications^17,22–24^. Fibrin is a tailorable hydrogel system utilizing fibrinogen, thrombin, and Factor XIIIa. Fibrinogen, a soluble 340-kDa clotting factor, is enzymatically converted, in the presence of Ca^2+^, into fibrin monomers by the protease thrombin^25^. These fibrin monomers undergo self-assembly and lateral aggregation to form protofibrils that are packed into fibers forming branched fibrous networks^25^. Factor XIIIa promotes the formation of covalent bonds between fibrinogen peptides to form a mesh network of fibrin fibers^22^. The fibrous network and mechanical properties of fibrin can be tuned by altering the composition ^26^. For instance, higher concentrations of factor XIIIa result in increasing the stiffness of fibrin by catalyzing fibrin covalent crosslinking and compacting fibers^27^. Moreover, fiber diameter and length are inversely proportional to thrombin concentration^22^, whereas increasing factor XIIIa concentrations lead to increased packing of protofibrils within the fibers^27^. Self-assembling peptide (SAP) hydrogels belong to the synthetic class and are produced using amino acids^28^. These peptides self-assemble to form nanofibrous hydrogels in physiological conditions. This self-assembly depends on the specific amino acid sequence of the peptide. These scaffolds consist of > 99% water, with fibers thought to be around 10 nm in diameter and 5–200 nm pores, closely mimicking the natural extracellular matrix (ECM)^28^. Arginine-alanine-aspartic acid-alanine-16 (RADA-16) is a member of the self-assembling peptide family, consisting of 16 residues, and can undergo self-assembly to form nanofibers by forming stable β-sheet structures in physiological saline, which in turn form an interwoven nanofibrous hydrogel^29^. The SAP (RADA-16) hydrogel has been used in several dental pulp tissue engineering studies with variable degrees of success^17,30,31^.

This study aimed to evaluate the effect of the inclusion of a novel macromolecule, chlorite-oxidized oxyamylose (COAM), on the microstructural properties of tailored fibrin and SAP hydrogels. COAM is a polyanionic polysaccharide derivative that acts as an antibacterial^32^ and antiviral agent^33,34^ and as an immunomodulator by interference with glycosaminoglycan (GAG) binding of chemokines^35^. Further goals were to assess the influence of the microstructural differences between the hydrogels on the in vitro behavior of hDPSCs and to identify the most suitable hydrogel for further in vivo experiments.

## Results

### COAM did not modify fibrin microstructure but affected SAP hydrogels leading to fiber aggregation

**Figure 1:**
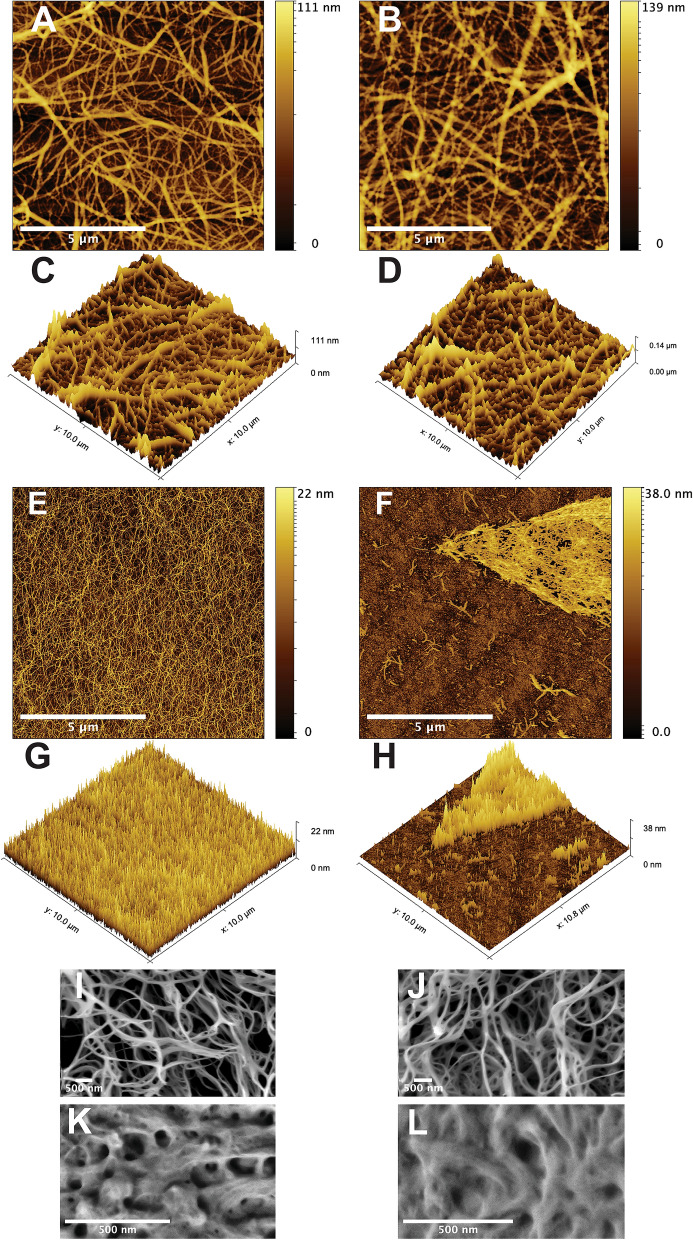
Hydrogel microstructure: Topographic atomic force microscopy (AFM) images of (**A**) fibrin hydrogel without COAM and (**B**) fibrin hydrogel with COAM. 3D AFM images of (**C**) fibrin hydrogel without COAM and (**D**) fibrin hydrogel with COAM. Topographic AFM images of (**E**) SAP hydrogel without COAM and (**F**) SAP hydrogel with COAM. 3D AFM images of (**G**) SAP hydrogel without COAM and (**H**) SAP hydrogel with COAM. Scanning electron microscopy (SEM) micrographs of (**I**) fibrin hydrogel without COAM, (**J**) fibrin hydrogel with COAM, (**K**) SAP hydrogel without COAM, and (**L**) SAP hydrogel with COAM. Color code scale in AFM images (**A**–**H**) represents fiber height. Scale bars in (**A**, **B**, **E**, **F**) = 5 µm, and in (**I**, **J**, **K**, **L**) = 500 nm.

**Figure 2:**
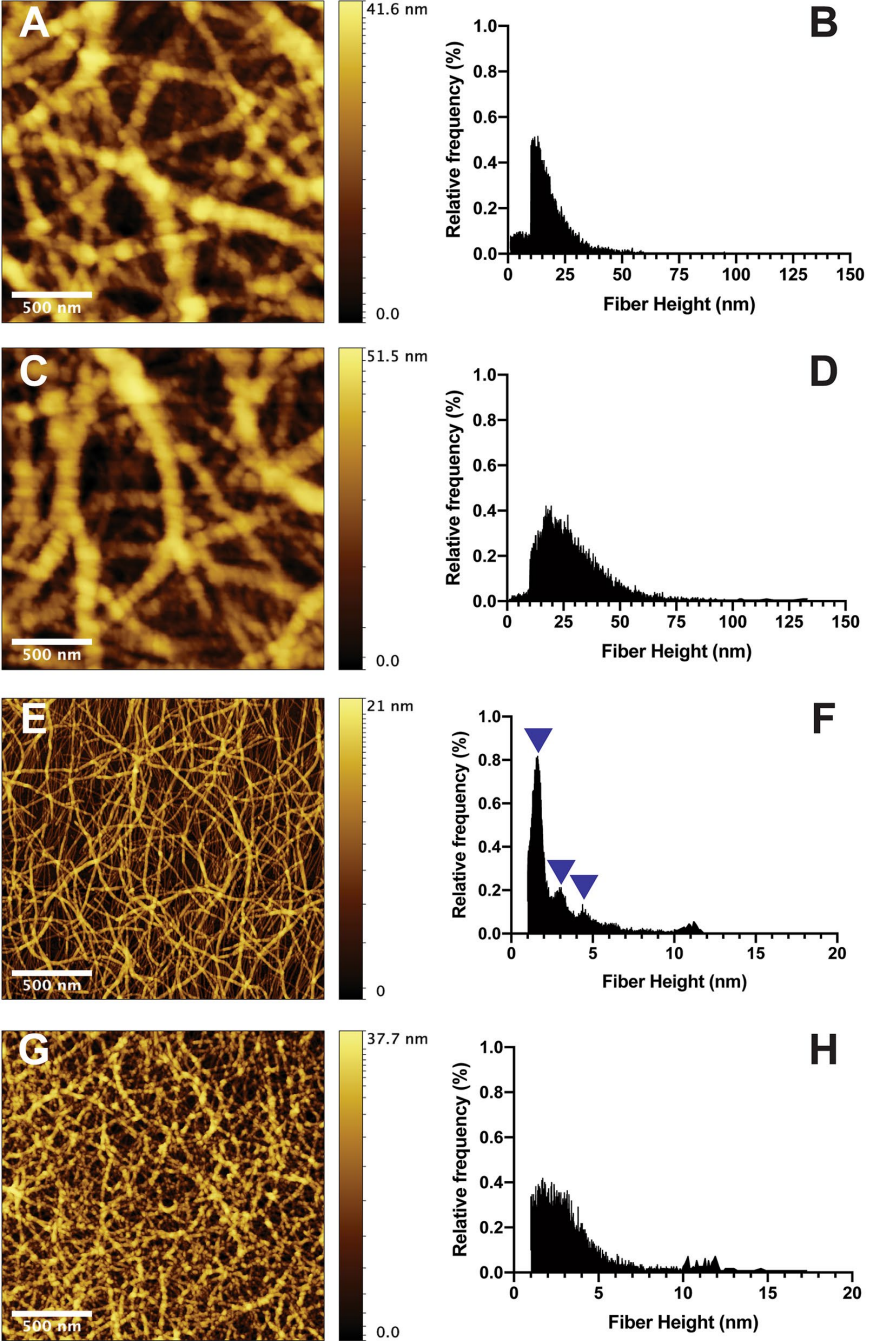
High-resolution atomic force microscopy (AFM) and quantitative fiber height distribution: (**A**, **B**) fibrin hydrogels without COAM, (**C**, **D**) fibrin hydrogels with COAM, (**E**, **F**) SAP hydrogels without COAM and with distinct peaks indicated by blue arrowheads, and (**G**, **H**) SAP hydrogels with COAM.

The inclusion of COAM did not alter the microstructure of the fibrin hydrogel at the fiber level as demonstrated by atomic force microscopy (AFM) images (Fig. 1A–D) and quantitative analysis (Table 1). However, SAP hydrogel microstructure (homogeneity) at the fiber level was affected by the inclusion of COAM (Fig. 1E–), leading to fiber aggregation (visible in Fig. 1F,H). SEM images further confirmed the microstructural stability of the fibrin hydrogels (Fig. 1I,J). In contrast, the effect of COAM inclusion on the morphology of the SAP hydrogels was not detected by SEM (Fig. 1K,L). The fiber height distribution showed no significant impact of COAM inclusion on fibrin hydrogels when comparing those without COAM (Fig. 2A,B) to those with COAM (Fig. 2C,D). SAP hydrogels without COAM (Fig. 2E,F) showed a distinct fiber height distribution with three peaks between 1 and 5 nm. After COAM inclusion (Fig. 2G,H), those three peaks disappeared, confirming aggregation at the fiber level.

**Table 1:** AFM image analysis using ctFIRE fiber extraction algorithm.

Parameter	Fibrin	Fibrin COAM	SAP	SAP COAM
Roughness average (Ra)	8.1 nm (SD: 1.6)	16.9 nm (SD: 7.5)	1.2 nm (SD: 0.1)	3.0 nm^a^ (SD: 0.4)
Fiber diameter	146.6 nm^b^ (± 1.1)	156.6 nm^b^ (± 1.2)	73.3 nm^b^ (± 0.3)	NA
Fiber length	1136.7 nm (± 50.2)	1077.7 nm (± 49.0)	1369.9 nm (± 24.7)	NA
Fiber straightness	0.93 (± 0.003)	0.93 (± 0.002)	0.80 (± 0.003)	NA
Fiber alignment	0.05	0.06	0.06	NA

*SD* Standard deviation.

± Standard Error of Mean.

^a^Measured on the aggregated fibers.

^b^Approximately overestimated by 15 nm because of tip diameter.

*NA* not available because fiber aggregation rendered measurements unreliable.

Topographic and quantitative microstructural analysis for the AFM images showed that both fibrin and SAP hydrogels have a nanofibrous structure at different scales (Table 1). The roughness average (Ra) was 8.1 (SD: 1.6) nm for fibrin hydrogels and 1.2 (SD: 0.1) nm for the SAP hydrogels. The inclusion of COAM in fibrin hydrogels increased the Ra to 16.9 (SD: 7.5) nm. However, this increase was not statistically significant (*p* > 0.05). The mean fiber diameters for the fibrin hydrogels were 146.6 ± 1.1 nm without COAM and 156.6 ± 1.2 nm with COAM (*p* > 0.05). The mean fiber diameter for the SAP hydrogels 73.2 ± 0.3 nm. For the SAP hydrogels with COAM, the fiber measurements were unreliable due to fiber aggregation (Fig. 1F,H); therefore, these measurements were not reported.

### Effect of COAM inclusion on fibrin and SAP hydrogel stiffness

**Figure 3:**
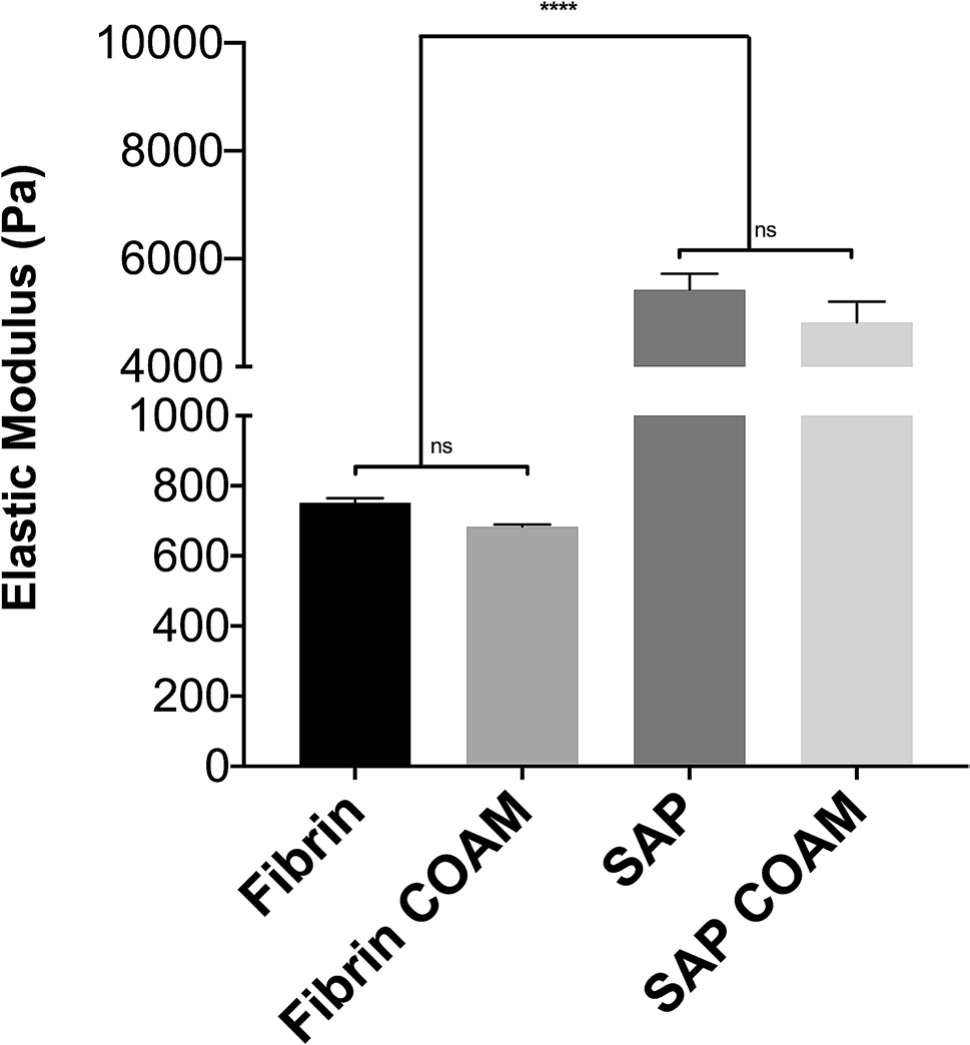
Hydrogels stiffness: The elastic modulus for fibrin hydrogels at 3.5 mg/ml fibrinogen concentration and SAP hydrogels at 3.5 mg/ml RADA-16 concentration. Results are presented as mean ± SEM; ns refers to not significant, *significant differences.

The elastic modulus for fibrin hydrogels at 3.5 mg/ml fibrinogen concentration was 752 ± 13 Pa before and 683 ± 6 Pa after the inclusion of COAM (Fig. 3). Furthermore, for the SAP hydrogels at 3.5 mg/ml RADA-16 concentration, the elastic modulus was 5425 ± 295 Pa before and 4821 ± 386 Pa after the inclusion of COAM (Fig. 3). The stiffness of the SAP hydrogels was sevenfold higher than the fibrin hydrogels (*p* < 0.05). The inclusion of COAM did not alter the stiffness of the fibrin and SAP hydrogels (*p* > 0.05).

### hDPSCs show higher viability and better attachment in fibrin hydrogels

**Figure 4:**
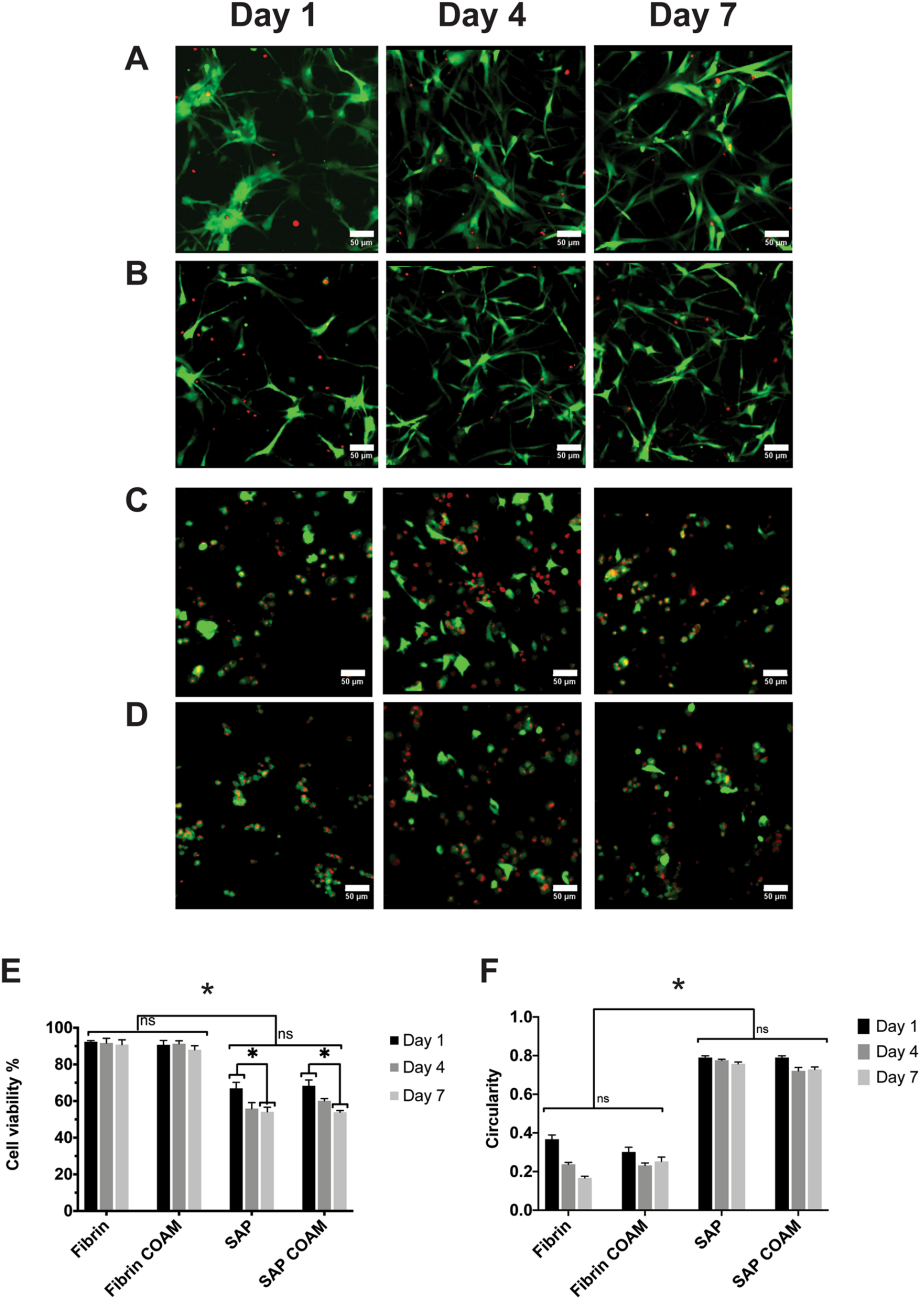
Cell viability (hDPSCs) in cell-laden hydrogels: representative Live/Dead images of hDPSCs encapsulated up to 7 days in (**A**) fibrin hydrogels without COAM, (**B**) fibrin hydrogels with COAM, (**C**) SAP hydrogels without COAM, and (**D**) SAP hydrogels with COAM. (**E**) Quantification of cell viability from Live/Dead images. (**F**) Cell shape (circularity) analysis from Live/Dead images using ImageJ. Green shows live cells, and red shows dead cells. Scale bars = 50 µm. Results are presented as mean ± SEM. ns refers to not significant, *significant differences.

According to an ANOVA analysis, we observed a significant effect on hDPSC viability in the maintenance medium for both the hydrogel type [F (1, 22) = 438.6, *p* < 0.0001] and the time period of the experiment [F (2, 22) = 9.9, *p* = 0.0008] (Fig. 4A–E). Post hoc comparisons using the Tukey test showed that viability did not significantly decrease over time for either the fibrin hydrogels without COAM or the fibrin hydrogels with COAM (*p* > 0.05). On the contrary, cell viability declined significantly in the SAP hydrogels without COAM from day 1 (66.9%) until day 7 (54.1%) and in the SAP hydrogels with COAM from day 1 (68.3%) until day 7 (53.9%) (*p* < 0.05) (Fig. 4E). The average hDPSC viability in the fibrin hydrogels was 91.3% without COAM and 89.9% with COAM over the 7-day test period (*p* > 0.05).

In addition, ANOVA analysis showed a significant effect on hDPSC circularity for the hydrogel type [F (1, 48) = 102.9, *p* < 0.0001] (Fig. 4F). hDPSCs adopted a spread morphology, indicated by a lower circularity value, in the fibrin hydrogels both with and without COAM (Fig. 4A,B). In contrast, hDPSCs remained rounded in the SAP hydrogels, again for the hydrogels both with and without COAM (Fig. 4C,D). These results indicate superior cell attachment in the fibrin hydrogels (Fig. 4A–D,F). Furthermore, the hDPSC morphology did not change significantly with time for any of the treatment conditions (Fig. 4F). The average circularity scores were 0.25 ± 0.05 in the fibrin hydrogels without COAM and 0.26 ± 0.02 in the fibrin hydrogels with COAM over the 7-day test period. The average circularity scores were 0.76 ± 0.01 in the SAP hydrogels without COAM and 0.74 ± 0.02 in the SAP hydrogels with COAM over the 7-day test period.

### DNA quantification to measure cell proliferation

According to an ANOVA analysis, there was a significant effect on the amount of DNA for both the hydrogel type [F (1, 49) = 89.85, *p* < 0.0001] and the presence of COAM [F (1, 49) = 12.47, *p* = 0.0009]. Post hoc comparisons using the Tukey test showed a significantly higher DNA content in response to the fibrin hydrogels with and without COAM compared to the SAP hydrogels with and without COAM (*p* < 0.05). hDPSCs showed a proliferative pattern in the fibrin hydrogels with an average 1.3-fold increase in DNA content at day 7 compared to day 0 for fibrin without COAM (*p* > 0.05) and a significant 2.1-fold increase for fibrin with COAM (*p* < 0.05).

**Figure 5:**
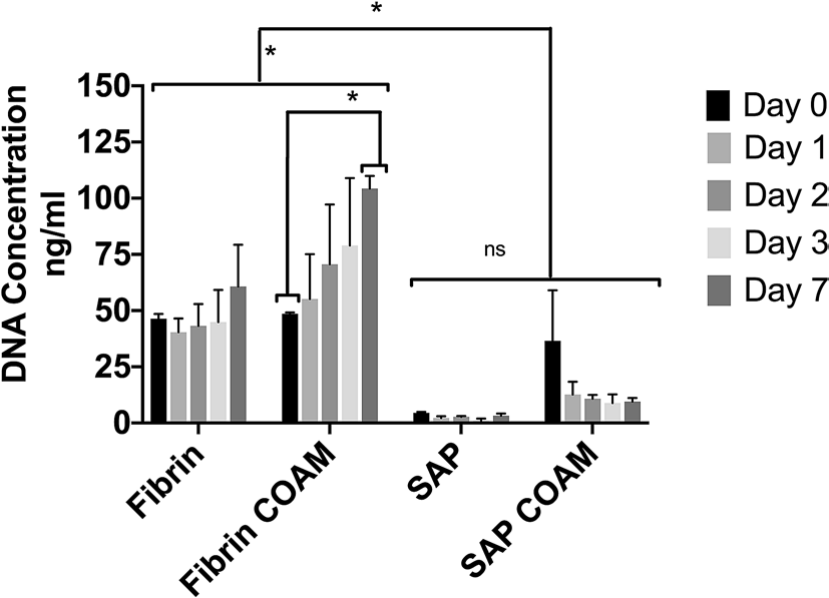
DNA content of cell-laden hydrogels: results are presented as mean ± SEM, ns refers to not significant, *significant differences.

A low DNA content was observed in the SAP hydrogels without COAM at day 0 with tenfold and eightfold lower DNA content compared to the fibrin hydrogels without COAM (*p* < 0.05) and SAP hydrogels with COAM (*p* > 0.05), respectively. SAP hydrogels with COAM showed higher DNA content than SAP hydrogels without COAM ranging between fivefold higher at day 1 and threefold higher at day 7. However, these differences were not statistically significant (*p* > 0.05). Moreover, the DNA content was stable in both SAP hydrogels from day 1 up to day 7 (Fig. 5).

## Discussion

In this study, we characterized the structural and mechanical characteristics of selected compositions of fibrin and SAP hydrogels. Nano- to micro-scale structural and mechanical cues are associated with biological responses in both native ECM and synthetic constructs^18^. Although the understanding of the association between the surface topography and the cellular response is still limited, it has been suggested that the nano-topography enhances cellular communication, such as in neural cell networks^36^, and protein adsorption, thus affecting the modulation of cellular interactions^37^. In this study, the nanoscale topographical features of fibrin and SAP hydrogels were different. The roughness average of the fibrin surface was sevenfold higher than that of the SAP hydrogel, along with a twofold increase in the average fiber diameter. Other features, such as fiber straightness and alignment, were comparable. Fibrin hydrogels showed structural stability after the inclusion of our experimental macromolecule, COAM, while SAP hydrogels were affected, leading to fiber aggregation. The effect of COAM inclusion on the morphology of the SAP hydrogels was not observed in the SEM images, which could be due to the sample preparation procedure that resulted in drying artifacts.

The measured stiffness of fibrin hydrogels, with the composition tested in the current study, was in the range of the stiffness of the native pulp tissue, which has previously been reported to be 800 Pa^38^. In addition, the stiffness of SAP hydrogels was found to be sevenfold higher than the fibrin hydrogels. hDPSCs are mesenchymal stem/stromal cells (MSCs) that pose the potential to differentiate into numerous cell types in vitro*,* including odontoblasts/osteoblasts, chondroblasts, adipocytes, and neuronal-like cells^13–15^. MSCs have been shown to specify lineage and commit to phenotypes with extreme sensitivity to tissue-level elasticity, as soft matrices induced neurogenic differentiation and stiffer matrices were osteogenic^39^. The current mechanical characterization results might aid in explaining previous observations of mineralized tissue formation within SAP (RADA-16) hydrogels encapsulating hDPSCs when they were implanted in an ectopic mouse model for 12 weeks^30^. Future studies should explore to which extent differences in matrix stiffness would affect hDPSC differentiation profiles in vivo.

The current study showed higher hDPSC survival in fibrin hydrogels compared to in SAP hydrogels. This agrees with the data reported previously^17^, where hDPSCs in fibrin hydrogels prepared at 10 mg/ml showed higher viability compared to hDPSCs in SAP (RADA-16) hydrogels when they were evaluated using an MTT assay. Moreover, other authors^40^ reported hDPSCs' survival at day 4 to be just above 60% in SAP (RADA-16) hydrogels at 1.5 mg/ml, reflecting the cell viability data reported in the current study. The higher cell survival in fibrin hydrogels can be likely explained by the presence of natural cell adhesion motifs^41^ facilitating cell attachment and elongated cellular morphology, which was demonstrated in the current study. In contrast, SAP hydrogels lack these cell adhesion motifs. Future studies could explore the possibility of improving cellular attachment to SAP hydrogels by conjugating bioactive short peptide motives such as the integrin-binding arginine-glycine-aspartic acid (RGD) to the C-terminus of the RADA-16 peptide. Another possible explanation for the low cell survival in SAP hydrogels could be the initial acidic pH (3.0) that is only neutralized after adding medium to induce gelation. The DNA quantification results confirmed differences in cell viability, with a tenfold lower DNA content measured at day 0 for SAP hydrogels compared to fibrin hydrogels. The DNA content in SAP hydrogels with COAM was eightfold and fivefold higher compared to SAP hydrogels without COAM at day 0 and day 1, respectively, suggesting an initial protective influence for COAM that needs to be further investigated. The hDPSC viability and the DNA content for the SAP hydrogels with and without COAM were then relatively stable over the remaining period of the experiments, strengthening the hypothesis that this drop in viability is related to the low attachment and the pH conditions at the time of encapsulation.

One interesting outcome was the effect of COAM on the increase of the DNA content, suggesting that the hDPSCs were proliferating more. Such effect for the presence of COAM may be explained in terms of a biological influence since no effect was observed for COAM inclusion on the structural and mechanical properties of the fibrin hydrogels. COAM is a polyanionic polysaccharide derivative with antibacterial properties^32^ and broad-spectrum antiviral activity that acts as an immunomodulator^33,34^. In previous studies, COAM has been shown to induce and bind chemokines such as granulocyte chemotactic protein-2 (GCP-2), leading to significant recruitment of myeloid cells in mice^42^. Furthermore, it has been demonstrated that COAM competes with GAGs for binding and recruitment of chemokines^35^. This COAM-chemokine binding complex influenced chemokine localization and the selectivity of leukocyte responses and migration^35^. hDPSCs and MSCs produce a plethora of soluble factors, cytokines, and chemokines influencing cellular growth, proliferation, migration, differentiation, and immune responses^43,44^. For example, insulin-like growth factor-1 (IGF-1), a cytokine produced by hDPSCs, was found to stimulate hDPSC proliferation in serum-free culture medium^45^. Moreover, hDPSCs overexpressing the chemokine stromal-derived factor-1 alpha (SDF-1a/CXCL12) showed higher cell proliferation compared to wild-type hDPSCs^46^. Therefore, one possible explanation for the higher DNA content in fibrin hydrogels with COAM could be the formation of a binding complex, increasing the availability of factors and chemokines involved in cellular proliferation inside the 3D hydrogel microenvironment. This is in line with preliminary experiments, in which we found that COAM binds SDF-1a/CXCL12, both on solid phase and in solution (unpublished data).

Finally, the current study presents a comprehensive structural and mechanical characterization for two promising biomaterials for dental pulp tissue engineering in combination with an analysis of biological features such as cell viability, shape, and proliferation. Future research will explore the influence of different hydrogel properties such as matrix stiffness on hDPSC migration and differentiation. Furthermore, the molecular mechanisms underlying the effect of COAM on hDPSC proliferation need to be investigated in detail in order to obtain insight to optimize their use in tissue engineering.

## Conclusion

The microstructural stability after the inclusion of COAM as well as the preservation of cell viability, elongated morphology, and higher DNA content observed in the fibrin hydrogels suggest this system as a promising carrier for COAM and application in endodontic regeneration.

## Materials and methods

All methods were performed in accordance with the relevant guidelines and regulations.

### COAM synthesis, hydrogel composition, and preparation

Chlorite-oxidized oxyamylose (COAM) was synthesized by two-step oxidation of amylose, purified, and fractionated according to molecular weight (MW) as described previously^33,47^. COAM was endotoxin-free and used as MW mixture (corresponding to protein molecular equivalent weights exceeding 100 kDa).

Fibrin hydrogels were prepared by mixing fibrinogen and thrombin components in equal volumes (pH 6.6), as described previously^22^. Plasminogen-depleted fibrinogen (Enzyme Research Laboratories, USA), derived from human plasma, was dissolved in 20 mM HEPES and 150 mM NaCl (fibrinogen buffer). Sterile stock solutions of thrombin (Sigma, USA), derived from human plasma, and factor XIII (Fibrogammin, CSL Behring, Germany) were prepared in 20 mM HEPES, 150 mM NaCl, 40 mM CaCl_2,_ and 0.1% BSA (thrombin buffer). Thrombin and factor XIII were mixed with the thrombin buffer and were kept in a water bath at 37 °C for 30 min to activate factor XIII to factor XIIIa. The control fibrin hydrogels were prepared at 3.5 mg/ml fibrinogen, 0.1 U/ml thrombin, and 0.1 U/ml factor XIII, whereas the test fibrin hydrogels were prepared at 3.5 mg/ml fibrinogen, 0.1 U/ml thrombin, 0.1 U/ml factor XIII, and 1 mg/ml COAM.

SAP RADA-16 hydrogels were prepared according to the manufacturer's instructions by mixing the peptide solution (PuraMatrix Peptide Hydrogel, BD Biosciences, USA) with 20% sucrose solution followed by adding an equal amount of phosphate-buffered saline (PBS) for gelation. The control SAP hydrogels were prepared at 3.5 mg/ml RADA-16 peptide, whereas the test SAP hydrogels were prepared at 3.5 mg/ml RADA-16 peptide and 1 mg/ml COAM.

### Structural and mechanical characterization

#### Atomic force microscopy (AFM)

AFM imaging was performed to characterize the microstructure of the different hydrogels at the fiber level. A 100 μl sample from each hydrogel composition (*n* = 3) was deposited on a silica sample holder and incubated at 37 °C for 30 min. After gelation, the hydrogel's top surface was carefully removed using gentle air blowing/drying to allow the imaging of the internal network. Agilent 5500 with MAC III controller and JPK Nanowizard 3 AFM systems were used for morphological imaging in intermittent contact mode in air. A sharp microlever probe MSNL-F (f = 120 kHz, k = 0.6 N/m, tip radius of curvature < 12 nm) was used. The AFM topography images were leveled, line-corrected, and measured (height and roughness profiles) using Gwyddion^48^. A fiber extraction algorithm, ct-FIRE^49^, was applied to the AFM images to characterize the fiber diameter, length, straightness, and alignment.

#### Scanning electron microscopy (SEM)

A 100 μl sample from each hydrogel composition (*n* = 3) was prepared then fixed using 4% glutaraldehyde in PBS for 30 min. This was followed by drying in an ethanol series for the fibrin hydrogels and freeze-drying for the SAP hydrogels because the SAP hydrogels disintegrated in ethanol. Subsequently, the samples were attached to aluminum stubs and sputter-coated with a 5 nm thick platinum layer under vacuum. The microstructure was then observed using an XL30 FEG scanning electron microscope (Philips, Panama).

#### Evaluation of hydrogel stiffness

The stiffness of hydrogels of each composition (*n* = *3*) was determined using a Chiaro Nanoindenter (Optics11, the Netherlands) by applying serial indentations with a spherical glass probe (r = 24.5 µm) attached to a flexible cantilever (k = 0.063 N/m). Loading and unloading velocities of the probe were set to 2 µm/s, with 2 s of holding period in between. For each sample, matrix scans (5 × 5 points) from three random locations were obtained. Load vs. displacement curves were extracted individually for each indentation point, and the Elastic Modulus (E) was calculated by using a Hertzian Contact Model (Poisson's ratio = 0.5) with Piuma Dataviewer Software (Optics11, the Netherlands), using Eq. (1):

1 $$F=\frac{4}{3}*E* \sqrt{R}*{h}^{3/2}*(1-{\vartheta }^{2})$$

where *F* is the applied force, $E$ is the elastic modulus, *R* is the radius of the probe, $h$ is the indentation depth, and $\vartheta$ is Poisson's ratio.

### Biological characterization

#### Primary cell cultures

Dental pulp tissues were acquired with informed consent from patients (15–20 years of age, male and female) undergoing extraction of third molars for therapeutic or orthodontic reasons as described previously^50^. Written informed consent was obtained from the patients or their parents, as approved by the medical ethical committee of Hasselt University, Belgium (protocol 13/0104U). The dental pulp tissue was harvested with forceps after mechanically fracturing the disinfected tooth with surgical chisels. Pulp tissues were then rinsed and transported at 37 °C in Eagle's Minimal Essential Medium, alpha modification (αMEM, Sigma-Aldrich, USA) supplemented with 2 mM l-glutamine (Sigma-Aldrich), 100 U/ml penicillin (Sigma-Aldrich), 100 μg/ml streptomycin (Sigma-Aldrich), and 10% fetal bovine serum (FBS, Gibco, Thermo Fisher Scientific, USA). hDPSCs were isolated according to the explant method and expanded in culture as described previously^50^. Cells were cultured in α-MEM, enriched with 10% heat-inactivated FBS (Biowest, Nuaillé, France), 2 mM l-glutamine, 100 U/mL penicillin, and 100 µg/mL streptomycin (Sigma-Aldrich). Only mycoplasma negative cells, screened with the PlasmoTest kit (InvivoGen), were used. All hDPSC cultures were tested for the expression of the following (stem) cell markers at the protein level by means of flow cytometry as described previously^50^: positive for CD29, CD73, CD90, and CD105 and negative for CD31, CD34, and CD45.

#### Evaluation of hDPSC viability

To obtain enhanced fluorescent protein (eGFP) labeled cells, pooled hDPSCs from three donors were transduced with a lentiviral vector encoding eGFP and a blasticidin resistance cassette. The selection was performed with blasticidin (10 µg/mL, InvivoGen, Toulouse, France). Stem cells were used until passage 15. These labeled hDPSCs at 1 × 10^6^ cells/ml seeding density were encapsulated in 100 μl hydrogels (*n* = 9) with and without COAM and deposited in a glass-bottom 96 well plate (CELLview Slide, Greiner, Austria). After gelation, an equal amount of maintenance culture medium was added (αMEM supplemented with 2 mM l-glutamine, 100 U/ml penicillin, 100 μg/ml streptomycin, and 1% FBS). Tranexamic acid at 0.5 mg/ml (Exacyl, Eumedica, Belgium) was added to the medium of fibrin hydrogels to prevent fibrin degradation. After 1, 4, and 7 days in culture, the nucleus of the cells was labeled with Hoechst 33342 (Invitrogen, USA), and the dead hDPSCs were labeled using the nucleic acid dye propidium iodide (PI) (Invitrogen) according to the manufacturer's instructions using an incubation time of 15 min at 37 °C. The images were collected using laser scanning confocal microscopy (LSM 880, ZEISS, Germany) using a 20 × objective (EC Plan-Neofluar 20 ×/0.50 M27). The fluorescence excitation/emission was measured at 490/552, 597/695, and 410/490 nm for GFP, PI, and Hoechst 33342, respectively. The number of live cells and dead cells were analyzed from 5 different regions per well (425 μm × 425 μm × 10 μm) in Fiji (Image J, National Institutes of Health, USA)^51^. Viability was calculated as a percent of live cells among the total number of live and dead cells.

#### Evaluation of hDPSC circularity (shape analysis)

Live cells from 5 different regions per well (425 μm × 425 μm × 10 μm) were segmented using a combination of watershed segmentation, thresholding, and manual contour correction for cell boundaries. Shape (circularity) of segmented cells per region, excluding cells on the image edges, was analyzed using the particle analysis plug-in in Fiji (Image J, National Institutes of Health, USA)^51^. The circularity score was averaged for each well, yielding a final circularity score ranging between 0 and 1, where the closer the score to 1, the closer the shape to a circle, which would indicate low cellular attachment.

#### Evaluation of hDPSC proliferation (PicoGreen/Quant-iT DNA Quantification)

hDPSCs at 5 × 10^5^ cells/ml seeding density were encapsulated in 100 μl hydrogels (*n* = 3 per gel and per time point) with and without COAM and deposited in a 96 well plate coated with 50 μl of the same hydrogel devoid of cells or COAM (TPP Tissue Culture Plates, Sigma-Aldrich). Hydrogels were prepared devoid of cells as blank replicates. In addition, 2D controls of 5 × 10^3^ cells were seeded in a 96 well plate. After gelation an equal amount of serum-free mesenchymal stem cell (MSC) medium (MesenCult-ACF Plus Medium, Stem Cell Technologies, Canada) supplemented with 2 mM l-glutamine, 100 U/ml penicillin, and 100 μg/ml streptomycin was added to each well. At baseline (day 0) and after 1, 2, 3, and 7 days in culture, a PicoGreen/Quant-iT kit (Invitrogen) was used to investigate the effect of different hydrogels on cellular proliferation. The DNA content of three hydrogels per condition and per time point was calculated for three independent experiments. Fibrin hydrogels were first digested in a buffer composed of 50 FU/ml nattokinase in 5 mM EDTA in PBS for 2 h at 37 °C. SAP hydrogels were digested in a buffer composed of 1 mg/ml Pronase (Thermo Fisher Scientific) in PBS for 2 h at 37 °C. The cells seeded in the control wells were released using Trypsin–EDTA. The contents of the wells were collected, and cell pellets were retrieved by centrifugation. Retrieved cell pellets were then lysed to extract DNA using 100 μl cell lysis buffer composed of 0.029% Sodium EDTA, 0.112% Sodium pyrophosphate decahydrate, 0.88% Sodium chloride, 0.315% Tris HCl, 1% Triton-X-100, 0.038% EGTA, 0.0001% Leupeptin, 0.019% Sodium orthovanadate, 0.0216% β-glycerophosphate and 1 mM PMSF (ab152163, Abcam) and centrifuged at 14,000 rpm at 4 °C to collect the supernatant. A 200 μl working solution representing each well (hydrogel) was prepared and aliquoted directly into black 96-well plates (Chimney well, Fluotrac, Greiner, Austria), according to the manufacturer’s instructions, and incubated for 5 min protected from light at room temperature. The fluorescence excitation/emission was measured at 481/520 nm using a microplate reader (Varioskan, Thermo Fisher Scientific). A standard curve was performed with λDNA, provided with the kit, and treated equally to the sample plates. The standards ranged from 10 ng/ml to 1 μg/ml λDNA and were used to calculate the final DNA content per ml of the sample.

### Statistical analysis

Statistical analysis was performed using the statistical software package GraphPad Prism 8.00 (GraphPad Software, La Jolla California USA). Comparison of the fiber measurements from AFM images was performed using a one-way analysis of variance (ANOVA). Comparison of the stiffness of the hydrogels was performed using a two-way ANOVA. The influence of the different experimental conditions and the time factor on cell viability, shape, and DNA quantity was modeled using a three-way ANOVA. All ANOVA tests were followed by Tukey's correction for multiple comparisons. Statistical significance was determined at *p* < 0.05. Descriptive statistics are represented as mean and standard deviation (SD), or standard error of the mean (±), where appropriate.
